# Cloning of HSP90, expression and localization of HSP70/90 in different tissues including lactating/non-lactating yak (Bos grunniens) breast tissue

**DOI:** 10.1371/journal.pone.0179321

**Published:** 2017-07-17

**Authors:** Penggang Liu, Sijiu Yu, Yan Cui, Junfeng He, Chuan Yu, Zexing Wen, Yangyang Pan, Kun Yang, Liangli Song, Xue Yang

**Affiliations:** College of Veterinary Medicine, Gansu Agricultural University, Lanzhou, Gansu, China; Technische Universitat Munchen, GERMANY

## Abstract

The aim of this study is to investigate the expression and localization of HSP70/90 in different tissues and explore the regulation effects of HSP70/90 at lactation period of female yaks. HSP90 mRNA was cloned from the heart samples of female yaks, Quantitative real-time (qRT-PCR), Western blotting (WB), immunohistochemistry and immunofluorescence assays were utilized to analyze the expressions of HSP70/90 mRNA and protein in different tissues. Sequence analysis showed that HSP90 is a conserved molecular chaperone of female yaks. The qRT-PCR, WB results showed that the expressions of HSP70/90 mRNA and protein were significantly different in different tissues, and 3-fold higher expression during the lactation period than the non-lactation period of breast tissue (*P* < 0.01). Immunohistochemistry and immunofluorescence assays results showed that HSP70/90 were located in the cardiac muscle cells, cerebellar medulla, theca cells lining at the reproductive system, and the mammary epithelia of the breasts. In addition, the expression level of HSP70 was higher than those of HSP90 in all examined tissues. Therefore, our results strongly suggest that the expression and localization of HSP70/90 could provide significant evidence to further research in tissue specific expression, and lactation function of female yaks.

## Introduction

Yak (Bos grunniens) is a special animal and one of the rare terrestrially cattle of China, which including primitive yak and modern yak. Yak is mainly distributed in the southwest of China, and yaks live in the cold and anoxic environment, especially in the altitude is 3000 meters high. It is known that yak has a prestigiously referred to as ‘the boat of the plateau’ [1]. Yak can usually adapt to the harsh environments, especially in protecting themselves against plateau hypoxia, cold stimulation, disease threats, etc., which are the main foci of attention and curiosity for many zoologists and medical scientists. It is reported that the proteins of the heat shock protein (HSP) family are involved in the environmental adaptability of these creatures. Thus, in this study, we focused on the impacts of HSP70/90 on yaks’ tissue specificities and lactation function.

In recent years, a growing number of studies showed that plenty of HSPs were identified, namely, ubiquitin, HSP27, HSP40, HSP60, HSP70, HSP90 and HSP110, meanwhile, the important biological functions of these small basic molecules have been elucidated [2]. HSP70/90 are found to protect the body mainly by increasing its quantity and spatial structure when encountering external stimuli and environmental changes [3], and they directly or indirectly participate in tumor occurrence, immune system function, wound healing, cell differentiation and transshipment of growth and development, but also the protection of cells from damage, organ failure, regulation of animal reproductive cell activity and protein expression. In short, HSP70/90 play coherently regulatory role to enhance the cells’ self-defense and maintain basic functions in the body [4, 5]. Previous studies have demonstrated that high concentrations of HSPs are involved in developing tissues and tumor cells such as stomach cancer, colon cancer, liver cancer, breast cancer and lung cancer cells. The expression levels of HSP70/90 are related to these cancers increased significantly, and they are highly expressed in leukemia and SLE patients, which explains why HSP70/90 play important role in the occurrence and development of various diseases [6, 7].

HSP70/90 mRNA and protein expression levels also show the corresponding changing trend with changes of temperature, sunshine intensity and environmental conditions as the animals’ body adapt to its environment. It was reported that a high density of locusts in the living environment, the science of animal behavior and HSP expression levels show close correlations [8]. HSP70/90 also can regulate the adaptability in the high temperature and low salinity environment in *Apostichopus japonicus* Selenka [9]. HSP27/HSP70 are powerfully proved to affect the time and cause of death when a body is on fire [10]. HSP70 can be used to contribute and protect the bodies of Boer goats when they are stimulated by high temperature [11].

HSP70/90 play a protective role during the cell division and embryonic developmental stage when the body is stimulated. The expression level of HSP70 has indicated that the temperature adaptability of zebu dermal fibroblasts is stronger than those of the hybrid cattle [12]; HSP70 induction by animal COD is higher in stained cystic follicles than others in the same category as well as in follicles, granulosa cells and sheath cells. The expression level of HSP90 in granulosa cells and the three stages of follicles are higher in the experimental group than the control group [13]. Fibroblasts in the pig fetus increased significantly with cold stimulation, and gradually reduced to normal level of HSP90 [14]. The expression level of HSP90 firstly increased and then decreased to normal levels, when boar sperm and pig oviduct epithelial tissue cells cultured in vitro were stimulated [15]. The inhibition of HSP90 not only stimulated the cryopreservation of equine spermatozoa but also induced the acrosome reaction by the phosphorylation of tyrosine and progesterone [16]. A lack of HSBP1 in the early embryonic development of rats and zebrafish led to the decrease of follicles and the disorder of the ectoderm and endoderm [17]. HSP60/70/90 have been demonstrated that can promote bone marrow dendritic cells to consume cell death debris in the bone membrane, and they are also important markers for receiving antigen stimulation and activation of the early immune system [18].

Previously, the unique characteristics of the anatomy of the internal genital organs and the ovarian morphology in yaks of different ages have been researched [19, 20], and the researcher have observed the histological structure of the yak uterus, ovaries and oviduct during the estrus cycle [21–23]. Follicular cells and oocytes during the development of the follicle in yaks are similar with other mammalians [24]. However, there are little existing information on the protein expression levels of HSP70/90 for yaks. In this study, we investigated the effects of HSP70/90 on the reproductive system of female yaks during lactation and non-lactation periods based on the following methods, tissue isolation, expression in vivo and identification of differences in various tissues and organs. Hence, our objectives are to investigate the expression and localization of HSP70/90 in the non-reproductive and reproductive systems of yaks.

## Materials and methods

### 1. Amplifying and sequencing the complete sequence of HSP90

The yaks were sampled from the Tibetan plateau in a Qinghai region pasture, Qinghai, China [25]. This study was approved by the Animal Ethics Committee of Gansu Agricultural University. A total of 16 healthy adult yaks (8 in the lactation period and 8 in the non-lactation period) were included in this study. All animals were kept under the same natural conditions (altitude: approximately 2300 meters, temperature: 2–5°C and oxygen content: 14.97%) [1]. We isolated total RNA from yak tissues using the TRIzol kit (R1100, USA) and conducted reverse transcription polymerase chain reaction (RT-PCR) to generate cDNA clones. The degenerate primers are used to amplify the HSP90 sequence based on the published partial sequence of HSP90 mRNA in the National Center for Biotechnology Information (NCBI) database (accession number NM-001012670.2). Primers for cloning the initial fragment of HSP90 mRNA were designed according to the prediction of conserved sequences in other *Bos taurus* animals (Table 1). The amplified segments were inserted into the cloning vector pMD-18T and transferred into *Escherichia coli* JM109 competent cells. The primers for 5’ HSP90 and 3’ HSP90 were designed by using the sequencing data, then the segments from 5’ to 3’ HSP90 from the first-strand cDNA were to solve the problem of clone and sequence.

**Table 1:** Primer sequences of target and house-keeping genes.

Primer name	Sequence(5′→3′)	*T*m (°C)	Note
Hsp90-1R	GATGAATACTCTGCGAACATACAA	56.9	RT-PCR
Hsp90-1F	ATGCCCGAGGAGACCCA	58.6	1113bp
Hsp90-2R	TGTTTGCTGTCCAGCCGTAT	58.9	RT-PCR
Hsp90-2F	GGAGGAGCGGAGAATAAAGG	58.2	1238bp
Hsp90-3R	CCCGATGTATGGACAATGACTC	59.2	RT-PCR
Hsp90-3F	GAAAGTTGAAAAGGTGGTTGTG	56.8	979bp
β-actin-F	GACCCAGATCATGTTTGAGACC	58.0	RT-PCR
β-actin-R	ATCTCCTTCTGCATCCTGTCAG	58.0	598bp
Hsp70-R	GCCTTGGTCTCCCCTTTGTAG	58.0	RT-qPCR
Hsp70-F	GCTGAACCCGCAGAACACG	58.0	158bp
Hsp90-R	GCTGAATAAAACCCGACACCA	62.0	RT-qPCR
Hsp90-F	CAAGCAAGATCGAACCCTCAC	62.0	174bp
β-actin-R	GCTCGGCTGTGGTGGTAAA	59.0	RT-qPCR
β-actin-F	AGGCTGTGCTGTCCCTGTATG	59.0	207bp

### 2. Analysis of HSP90 sequence

The ORFs in the complete mRNA sequence of HSP90 were identified by using the ORF finder (http://www.ncbi.nlm.nih.gov/projects/gorf/orfig.cgi), and then the nucleotide sequences were translated into amino acids using Vector NTI 11 software [26]. The HSP90 sequence was analyzed by using ODC HSP90 finder software, and the codon region and frameshifting site were identified [27].

Homology searches were performed by using BLASTn and BLASTp in the NCBI database. The Conserved Domain (CD) Search was used to identify the CDs in the predicted protein sequences (http://www.ncbi.nlm.nih.gov/Structure/cdd/cdd.shtml). The 3-D structure of the protein was predicted according to the methods described in the website (http://bioinf.cs.ucl.ac.uk/psipred/). The deduced amino acid sequence of HSP90 was aligned by using CLC main workbench software (http://www.clcbio.com) with the known homologous proteins of the HSP90 class obtained from GenBank. A phylogenetic tree was constructed by using CLC main workbench software, and the neighbor-joining method with the amino acid sequences of HSP90 from the SwissProt databank/GenBank.

### 3. Gene expression of HSP70/90 in different tissues

The HSP70/90 tissue distribution in yaks were detected during lactation and non-lactation periods in various organs including the non-reproductive system (kidney, heart, brain, liver, lung and spleen) and the reproductive system (uterus, oviduct and ovary). Eleven tissues were dissected and collected in frozen liquid nitrogen, and then kept at -80°C before using. The total RNAs from various tissues were extracted by using the TRIzol reagent. The expression levels of HSP70/90 in the different tissues were identified by using quantitative qRT-PCR (Invitrogen, USA) with HSP70/90 specific primers. Actin was used as a reference gene to normalize the amount and quality of each cDNA as this gene was expressed constitutively in the different tissues [28].

### 4. Protein expression and activity determination of HSP70/90

Tissue samples were selected from healthy adult yak subjects. Frozen samples were ground in liquid nitrogen, and transferred to centrifuge tubes with RIPA/PMSF (Solarbio, China). After fully blending and mixing to a pink color, sample tubes were incubated on a spiral oscillator for 2 hours (200 r/h) on ice. After centrifuging at 4°C for 10 min (12000 r/h), we obtained the complete divided protein. In addition, we measured the total protein concentration for each sample. The total protein concentration was then adjusted to the same level, and 4× sample buffer at 100°C was added for 10 min to completely denature the protein. Aside from these samples, eleven tissue samples were kept at -80°C before using.

Eluted protein was separated into beads by using a spin column (Bio-Rad), and separated on a 5% SDS-PAGE gel for WB. After electrophoresis, proteins were transferred from the gel onto NC membranes (Millipore Corporation, Billerica, MA, USA). The membranes containing protein were blocked with 5% fat-free milk in TBST at room temperature for 2 h and hybridized using HSP70/90 Abcam (1:1000) or rabbit polyclonal Abcam (1:2000) at 4°C overnight. The membranes were then washed 5 times with TBST and labeled with HRP-conjugated secondary Ab (1:4000) for 2 h at room temperature. After washing 5 times with 1× TBST, HSP70/90 were detected on the membrane with the ECL detection kit (Beyotime, China). The intensities of the bands on the blots were measured by using a densitometric analysis system (Bio-Rad). The intensities of the β-actin bands were used for normalization.

### 5. Immunofluorescence and immunohistochemical assays

The main organs and tissues of the yaks were fixed in 4% paraformaldehyde solution at room temperature for hebdomad. Tissue pieces were clipped and paraffin embedded, and then the sections were sliced, dried and saved.

For immunohistochemical staining to investigate HSP70 and HSP90 expression levels, samples were dewaxed by using dimethyl benzene and then dehydrated with an ascending alcohol gradient. The sections were rehydrated, and the antigen was repaired with 3% deionized H_2_O_2_ (15–20 min) and sealed with goat serum (15–20 min). After overnight incubation at 4°C with the primary rabbit anti-HSP70 and mouse anti-HSP90 monoclonal antibodies (1:300, Abcam, Hong Kong), the sections were then incubated with the secondary antibody. The labeled samples were then counterstained with 3–3'-diaminobenzidine [29]. Nuclei were complex and preserved. There were differences in immunofluorescence between the two incubations, and there was no need to re-dye the nuclei.

### 6. Measurement and statistical analyses

Intensity measurements for the WB images and the immunofluorescence and immunohistochemical assays were performed using integrated optical density and measured by Image-Pro plus 6.0. All data were analyzed using SPSS 21.0. The Spearman Correlation of Coefficients were analyzed between β-actin and sample protein levels. The other data were analyzed by one-way ANOVA and Duncan’s post hoc test. P-values between groups less than 0.05 were regarded to be statistically significant [30].

## Results

### 1. Analysis of the HSP90 cDNA sequence and the deduced amino acid sequences

The cDNA sequence of HSP90 was cloned and submitted to GenBank with the accession number KF690730.1.

The nucleotide sequence of HSP90 was 3066 bp. By analyzing this contig, the predicted HSP90 cDNA of 3066 bp contains an open reading frame (ORF) of 2166 bp, which is from 179 to 2347. The first codon was ATG, and the stop codon was TGA. The analysis of the HSP90 cDNA sequence was confirmed by these predictions in this study. To verify, three primers, H90-1, H90-2 and H90-3, were used to clone the full-length HSP90 ORF. Using RT-PCR analysis, a cDNA fragment of 2166 bp was successfully isolated from bovine heart total RNA. This confirmed cDNA sequence was deposited in GenBank with accession number KF690730. To obtain the genomic sequence of HSP90, the publicly available cow genome database through the NCBI Bovine Genome Resources (http://www.ncbi.nlm.nih.gov/projects/genome/guide/cow/) was queried by using the HSP90 cDNA sequence. A cow (*Bos taurus*) contig (GenBank accession no. NM_001012670) that encompassed the entire HSP90 gene was identified using BLASTGen analysis [31].

Analysis of the basic physical and chemical properties of HSP90 showed that the protein coding region atomic number is 11710. The molecular formula is C_3674_H_5859_N_971_O_1179_S_27_, the molecular weight is 83363.3 D, and the theoretical isoelectric point (pI) is 4.897. The half-life of Hsp90 protein is approximately 30 hours, the instability index is 41.12, the fat soluble index is 80.33, and the average hydrophobicity index is -0.722.

**Fig 1:**
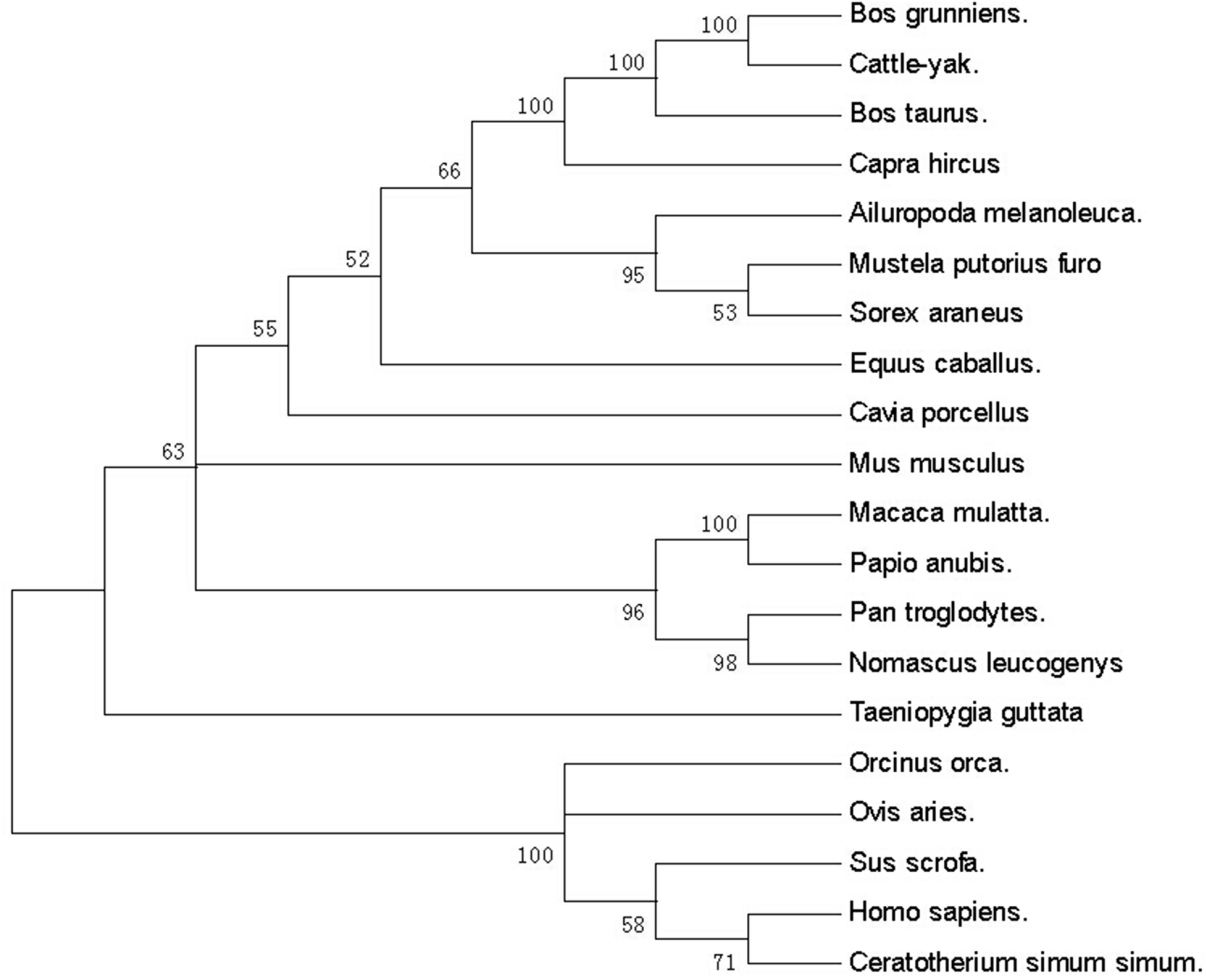
The HSP90 phylogenetic tree of Bos grunniens. The phylogenetic tree was constructed with CLC main workbench software using the neighbor-joining method for the amino acid sequences of HSP90 from the Swiss-Prot databank/GenBank.

A molecular phylogenetic tree was constructed to analyze the evolutionary relationship of the HSP90 amino acid sequences (Fig 1). The tree showed that the yak HSP90 evolutionarily shared a higher sequence identity with cattle-yak, *Bos taurus*, *Capra hircus*, *Ailuropoda melanoleuca*, *Mustela putorius furo*, *Sorex aranens*, *Equus caballus* and *Cavia porcellus*.

### 2. Analysis of the deduced proteins

The predicted amino acid sequence of HSP90 was compared with the known *Bos taurus* sequences using BLASTp. The yak HSP90 protein sequence shared a low percentage of similarity to other known HSP90 protein sequences. This result indicated that the HSP90 proteins were different from others in the HSP family. The HSP90 amino acid sequence index of similarity between the yaks and *Bos* cattle-yak was approximately 99.56%, and that between the yaks and *Bos taurus* was 99.33%, with approximately 98.35% and 98.34% identity to the HSPs of *Ovis aries* and *Sus scrofa*, and 98.29% identity to the HSPs of humans. The highest level of similarity appeared near the C-terminus, and the similarity in the N-terminus and the middle of the amino acid sequence was very low.

Nucleotides with variability led to differences in amino acids, such as E to G, R to Q, AAU to ATV, and T to M. The most important outcome of this variance is that it led to changes in the protein spatial structure and caused the differences in protein function. SDS-PAGE analysis indicated that the fusion protein HSP70 was approximately 70 kDa, whereas the fusion protein HSP90 was approximately 84.7 kDa.

### 3. Expression and distribution of HSP70 and HSP90 in yaks

#### 3.1 HSP70 and HSP90 expression in the non-reproductive system

**Fig 2:**
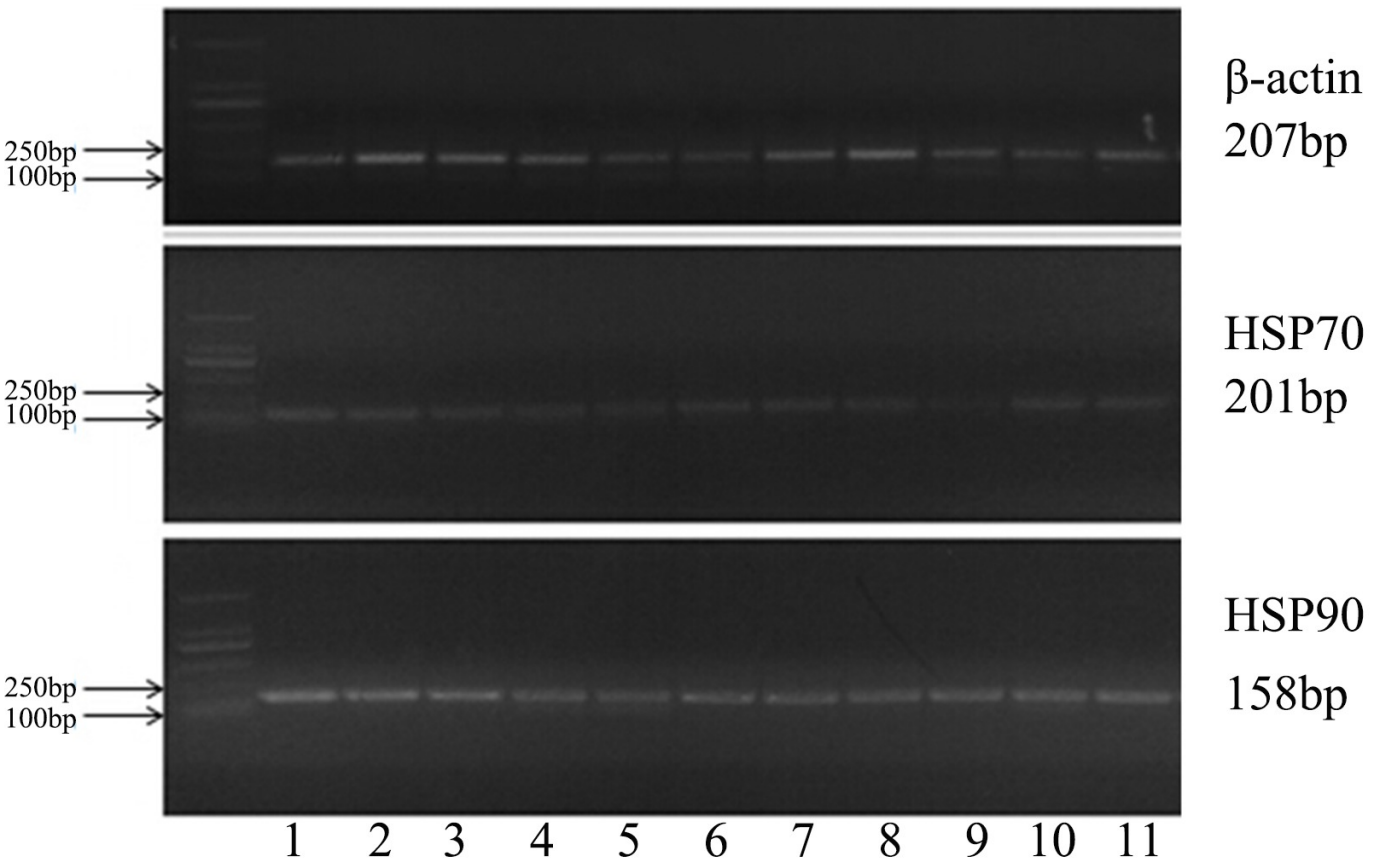
The results of RT-PCR. (1) kidney; (2) heart; (3) cerebellum; (4) liver; (5) lung; (6) spleen; (7) uterus; (8) oviduct; (9) ovary; (10) lactation period; and (11) non-lactation period.

**Fig 3:**
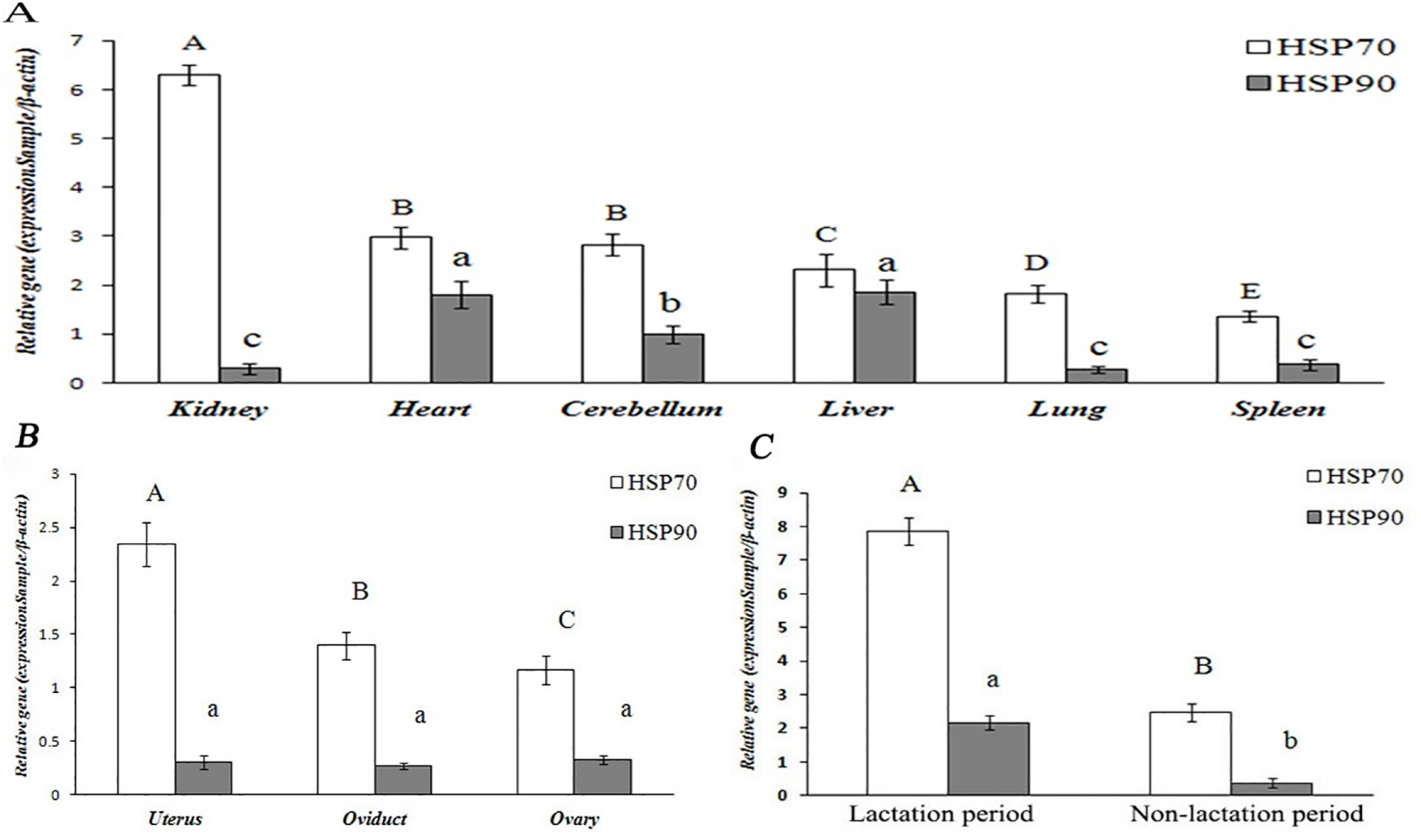
The gene expression levels of HSP70 and HSP90 in tissues of female yaks. **The results of real-time PCR**. (A) Non-reproductive system; (B) female reproductive system; and (C) breast at different lactation periods.

**Fig 4:**
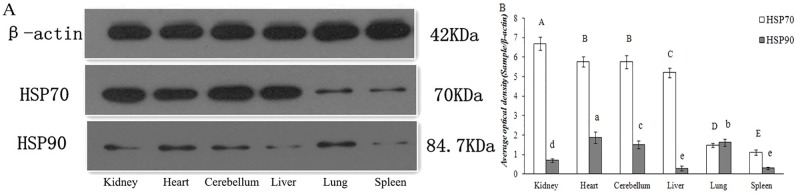
Protein expression levels of HSP70 and HSP90 detected using Western blotting analyses in the non-reproductive system of female yaks. (A) Schematic representation of bands and size. (B) Comparative analysis of relative expression by integrated optical density.

The tissue distribution of the yak HSP70/90 gene were investigated using qRT-PCR with the total RNA isolated from yak tissue as a template, as shown in (Fig 2). The HSP70/90 gene and protein were expressed at different levels in the non-reproductive system, including the heart, kidney, brain, liver, spleen, and lung, as shown in (Figs 3A and 4). The highest expression level of HSP70 was observed in the kidney, followed by the heart and brain. In contrast, the lowest expression level was in the spleen, followed by the liver, and lung. The highest expression level of HSP90 was observed in the liver, followed by the heart, brain and spleen. In contrast, the lowest expression level was in the lung, followed by the kidney. The expression levels of HSP70 were consistently higher than HSP90 in almost all the tested tissues (P<0.01), except in the kidney and cerebellum, as shown in (Table 2). The expression levels of HSP70 were higher than those of HSP90 in the heart, liver, lung, and spleen. However, the protein expression levels of HSP70 were slightly lower than those of HSP90 in the yak lung, as shown in (Table 3).

**Table 2:** RT-qPCR results of HSP70/90 gene in different tissues and organs of yak.

Tissues and Organs	ΔCT	ΔΔCT (ΔCT_sample_-ΔCT_ovary/oviduct_)	2^-ΔΔCT^
***HSP70***			
Kidney	6.3110±0.2525	5.1477±0.2525	0.0282
Heart	2.9800±0.2311	1.8167±0.2311	0.2838
Cerebellum	2.8240±0.2773	1.6607±0.2773	0.3163
Liver	2.3080±0.2694	1.1447±0.2694	0.4523
Lung	1.8220±0.2506	0.6587±0.2506	0.6334
Spleen	1.3651±0.1043	0.2018±0.1043	0.8695
Uterus	2.3448±0.2075	1.1816±0.2075	0.4409
Oviduct	1.3973±0.1304	0.2340±0.1304	0.8503
Ovary	1.1633±0.1370	0.0000±0.1370	1
Lactating period	7.8690±0.4023	6.7057±0.4023	0.0096
Non-lactating period	2.4594±0.2649	2.1945±0.2649	0.2185
***HSP90***			
Kidney	0.2924±0.2361	0.0265±0.2361	0.9818
Heart	1.8050±0.2159	1.5391±0.2159	0.3441
Cerebellum	0.9983±0.3165	0.7324±0.3165	0.6019
Liver	1.8620±0.1624	1.5961±0.1624	0.3308
Lung	0.2742±0.2155	0.0083±0.2155	0.9943
Spleen	0.3733±0.0942	0.1074±0.0942	0.9282
Uterus	0.3019±0.0607	0.0360±0.0607	0.9754
Oviduct	0.2659±0.0270	0.0000±0.0270	1
Ovary	0.3283±0.0395	0.0625±0.0395	0.9576
Lactating period	2.1720±0.2126	1.9061±0.2126	0.2668
Non-lactating period	0.3694±0.1451	0.1035±0.1451	0.9308

**Table 3:** Integrated optical density of *HSP70/90* expression in different tissues and organs of yak.

Tissues and Organs	Integral optical density (_IntDen/Area_)	Relative density (_sample/β-action_)
***HSP70***		
Kidney	70.1945±2.3398	6.6897±0.5850
Heart	67.9436±2.2648	5.7707±0.5662
Cerebellum	69.9062±2.3302	5.7537±0.5826
Liver	67.3054±2.2435	5.2088±0.5609
Lung	60.1482±2.0049	1.4834±0.2012
Spleen	48.9594±1.6320	1.1159±0.1080
Uterus	64.18532±2.1395	3.3083±0.5349
Oviduct	39.00248±1.3001	1.8917±0.2250
Ovary	27.47801±0.9159	0.3660±0.0929
Lactating period	71.73044±2.3910	6.7404±0.5978
Non-lactating period	37.90629±1.2635	2.1013±0.3159
***HSP90***		
Kidney	50.7714±1.6924	0.6983±0.2115
Heart	47.6372±1.5879	1.8703±0.1985
Cerebellum	45.1638±1.5055	1.4993±0.1882
Liver	42.7329±1.4244	0.2975±0.1781
Lung	41.4212±1.3807	1.6256±0.1726
Spleen	40.8789±1.3626	0.2990±0.1703
Uterus	42.6790±1.4226	2.6102±0.1779
Oviduct	20.7937±0.6931	0.8164±0.0866
Ovary	19.8312±0.6610	0.6209±0.0826
Lactating period	51.5472±1.7182	3.33199±0.2148
Non-lactating period	27.1803±0.9060	1.0127±0.1133

**Fig 5:**
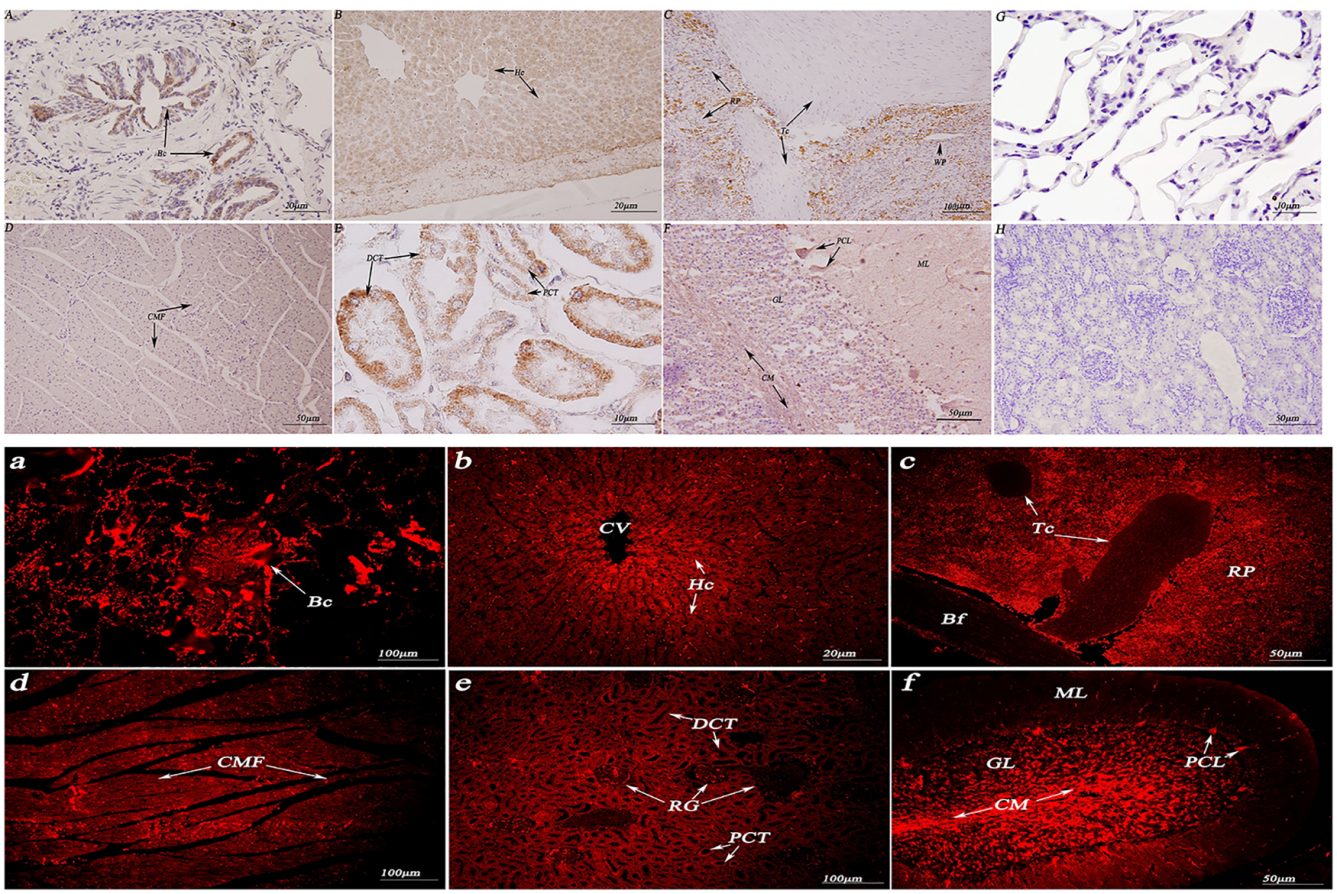
Immunohistochemistry and immunofluorescence localization of HSP70 in the non-reproductive system of female yaks. (A) Positive staining for HSP70 was observed in the terminal bronchioles of the lungs. (B) Positive staining for HSP70 was observed in hepatocytes of the *Bos grunniens* liver. (C) Positive staining for HSP70 was observed in the white pulp of the spleen. (D) Positive staining for HSP70 was observed in cardiac muscle fibers of the *Bos grunniens* heart. (E) Positive staining for HSP70 was observed in the distal convoluted tubule and proximal convoluted tubule of the kidney. (F) Positive staining for HSP70 was observed in the cerebellar medulla, the granular layer and the Purkinje cell layer of the cerebellum. (G) and (H) The control sections collected from the lungs and kidneys of *Bos grunniens*, respectively, without immunoreactions (negative control). Terminal bronchiole (TB); hepatocyte (Hc); central vein (CV); cardiac muscle fibers (CMF); distal convoluted tubule (DCT); Purkinje cell layer (PCL); renal glomerulus (RG); biofilm (Bf); trabecula (Tc); red pulp (RP); white pulp (WP); cerebellar medulla (CM); molecular layer (ML); granular layer (GL); and proximal convoluted tubule (PCT).

**Fig 6:**
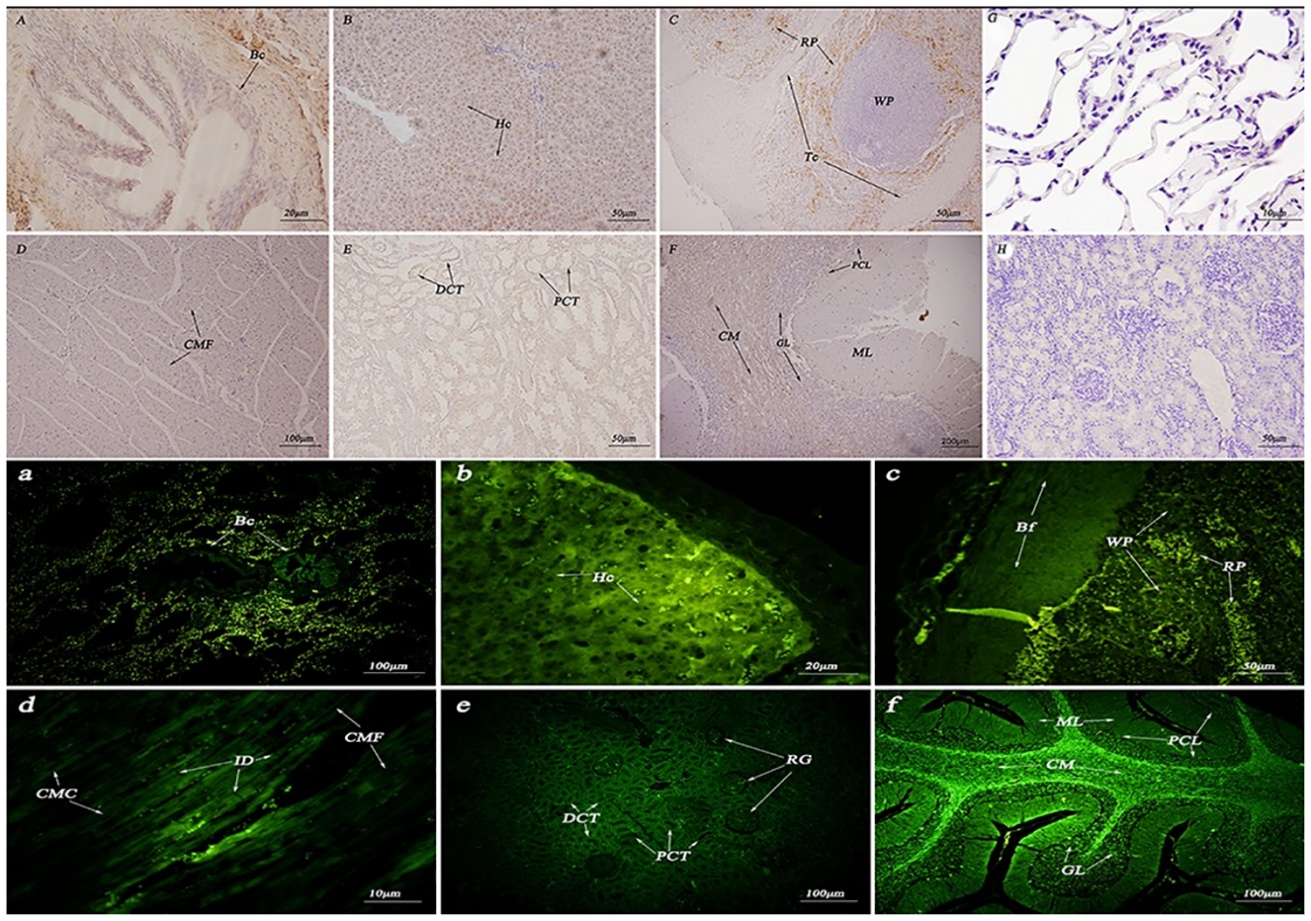
Immunohistochemistry and immunofluorescence localization of HSP90 in the non-reproductive system of female yaks. (A) Positive staining for HSP90 was observed in the terminal bronchioles of the lungs. (B) Positive staining for HSP90 was observed in hepatocytes of the liver. (C) Positive staining for HSP90 was observed in the red pulp of the spleen. (D) Positive staining for HSP90 was observed in the myocardial cell cytoplasm of the heart. (E) Positive staining for HSP90 was observed in the distal convoluted tubule and proximal convoluted tubule of the kidney. (F) Positive staining for HSP90 was observed in the cerebellar medulla and the granular layer of the cerebellum. (G) and (H) The control sections collected from the lungs and kidneys of *Bos grunniens*, respectively, without immunoreactions (negative control). Terminal bronchiole (TB); hepatocyte (Hc); central vein (CV); cardiac muscle fibers (CMF); cardiac muscle cell (CMC); intercalated disk (ID); distal convoluted tubule (DCT); Purkinje cell layer (PCL); renal glomerulus (RG); biofilm (Bf); trabecula (Tc); red pulp (RP); white pulp (WP); cerebellar medulla (CM); molecular layer (ML); granular layer (GL); and proximal convoluted tubule (PCT).

As shown in (Figs 5 and 6), HSP70/90 were mainly detected in kidney tubules, cardiac muscle cells, hepatocytes, Purkinje cells and the cerebellar medulla. Positioning analysis indicated that the HSP70/90 protein were mainly concentrated in the connective tissue and mammary epithelia. Thus, the proteins are mainly concentrated on the cell membrane and in the cytoplasm, but not in the nucleus.

#### 3.2. HSP70/90 expression levels in the reproductive system of female yaks

**Fig 7:**
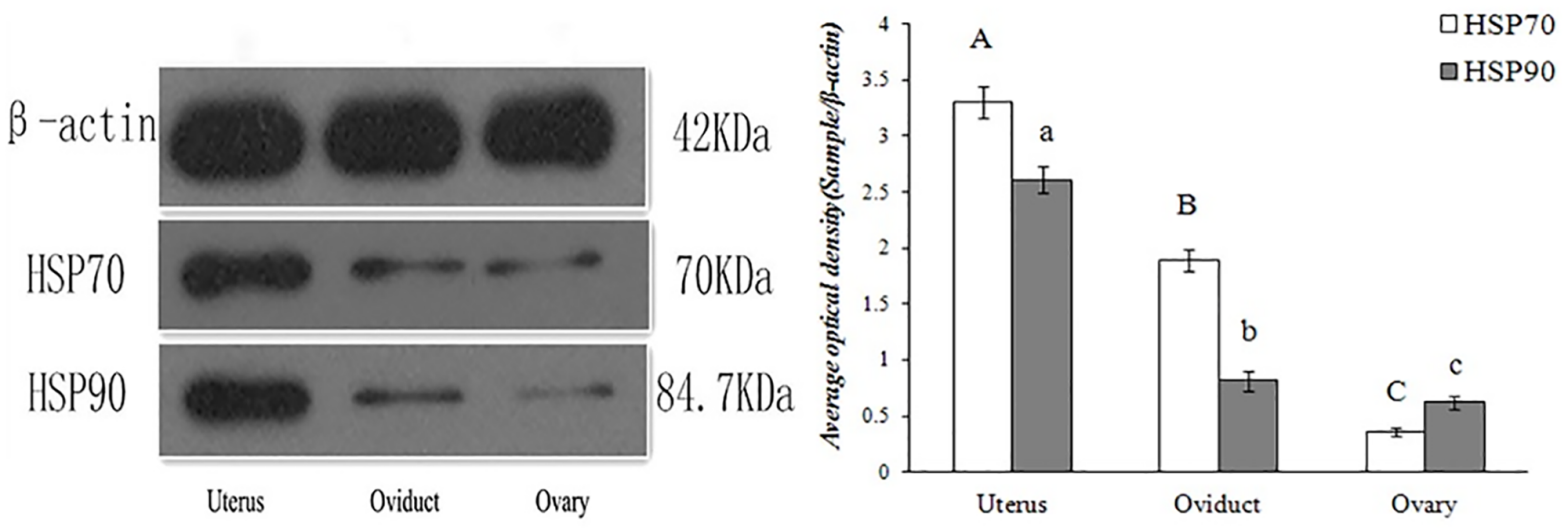
Protein expression levels of HSP70 and HSP90 detected using Western blotting analyses in the reproductive system of female yaks. (A) Schematic representation of bands and size. (B) Comparative analysis of relative expression by integrated optical density.

HSP70/90 expression levels showed significant differences in the female yaks’ reproductive systems (P<0.01). As shown in (Figs 3B and 7), the highest expression level of HSP70 was observed in the uterus, followed by the oviduct. In contrast, the lowest expression level was in the ovary. The highest expression level of HSP90 was observed in the ovary, followed by the uterus. In contrast, the lowest expression level was in the oviduct. However, the HSP90 gene expression levels were not significantly differences in the uteri, ovaries and oviducts of the yaks, as shown in (Tables 2 and 3).

**Fig 8:**
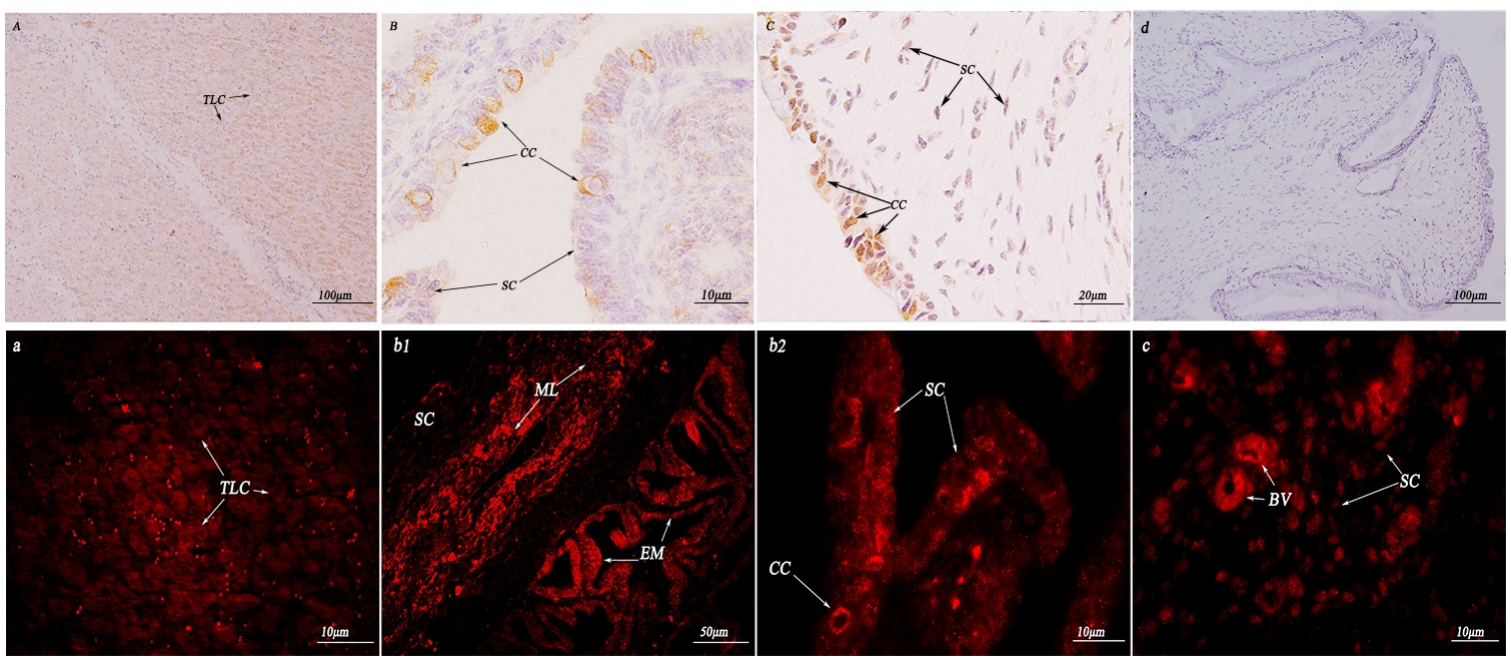
Immunohistochemistry and immunofluorescence localization of HSP70 in the reproductive system of female yaks. (A) Positive staining for HSP70 was observed in the theca lining cells of the ovary. (B1) and (B2) Positive staining for HSP70 was observed in the ciliated cells of the oviduct. (C) Positive staining for HSP70 was observed in the ciliated cells and stroma cells of the uterus. (D) The control section collected from the uterus of *Bos grunniens*, without immunoreactions (negative control). Theca lining cells (TLC); ciliated cell (CC); stroma cell (SC); mucous membrane (MM); muscular layer (ML); serous coat (SC); epithelium mucosae (EM); blood vessel (BV); and secretory cell (SC).

**Fig 9:**
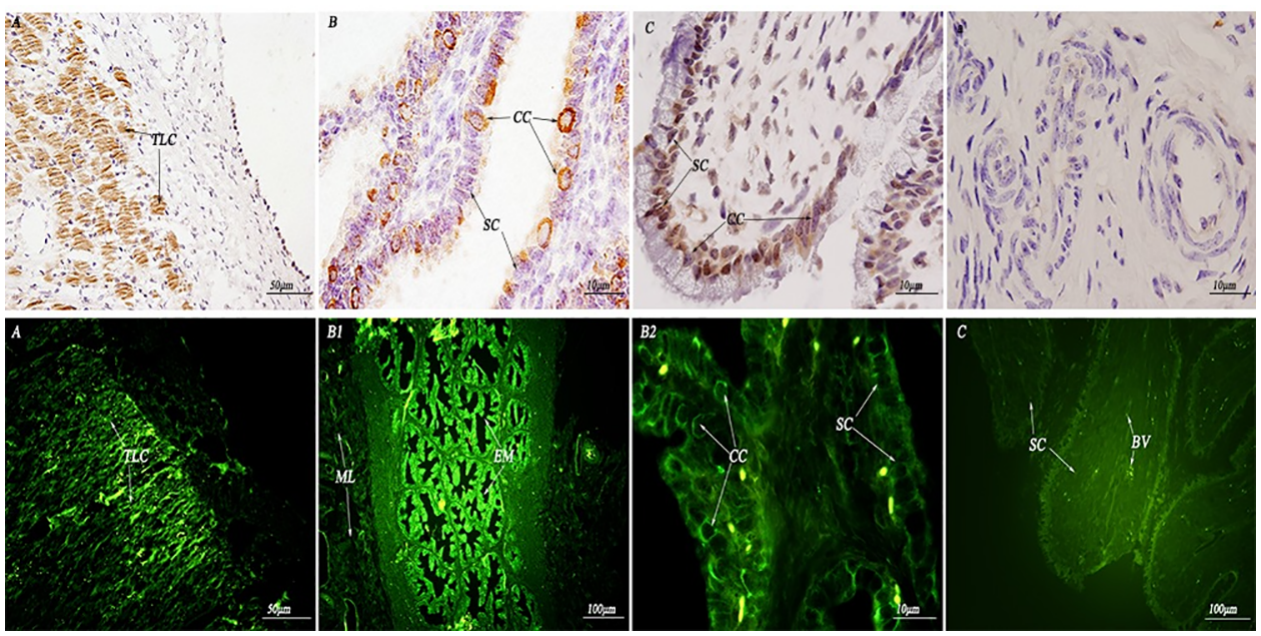
Immunohistochemistry and immunofluorescence localization of HSP90 in the reproductive system of female yaks. (A) Positive staining for HSP90 was observed in the theca lining cells of the ovary. (B1) and (B2) Positive staining for HSP90 was observed in the ciliated cells of the oviduct. (C) Positive staining for HSP90 was observed in the ciliated cells and stroma cells of the uterus. (D) The control section collected from the uterus of *Bos grunniens*, without immunoreactions (negative control). Theca lining cells (TLC); ciliated cell (CC); stroma cell (SC); mucous membrane (MM); muscular layer (ML); serous coat (SC); epithelium mucosae (EM); blood vessel (BV); and secretory cell (SC).

As shown in (Figs 8 and 9), this study mainly focused on the expression levels in the uterine endometrial epithelia and lamina propria plasma cell nuclei. The expression levels in the tubal mucosa plica epithelial ciliated cell membranes were the strongest. The expression levels in the ovary membranes and cortex were strong, and the expression levels in the cytoplasm and nuclei of connective tissue were the weakest.

#### 3.3. HSP70/90 expression levels in different lactation stages

**Fig 10:**
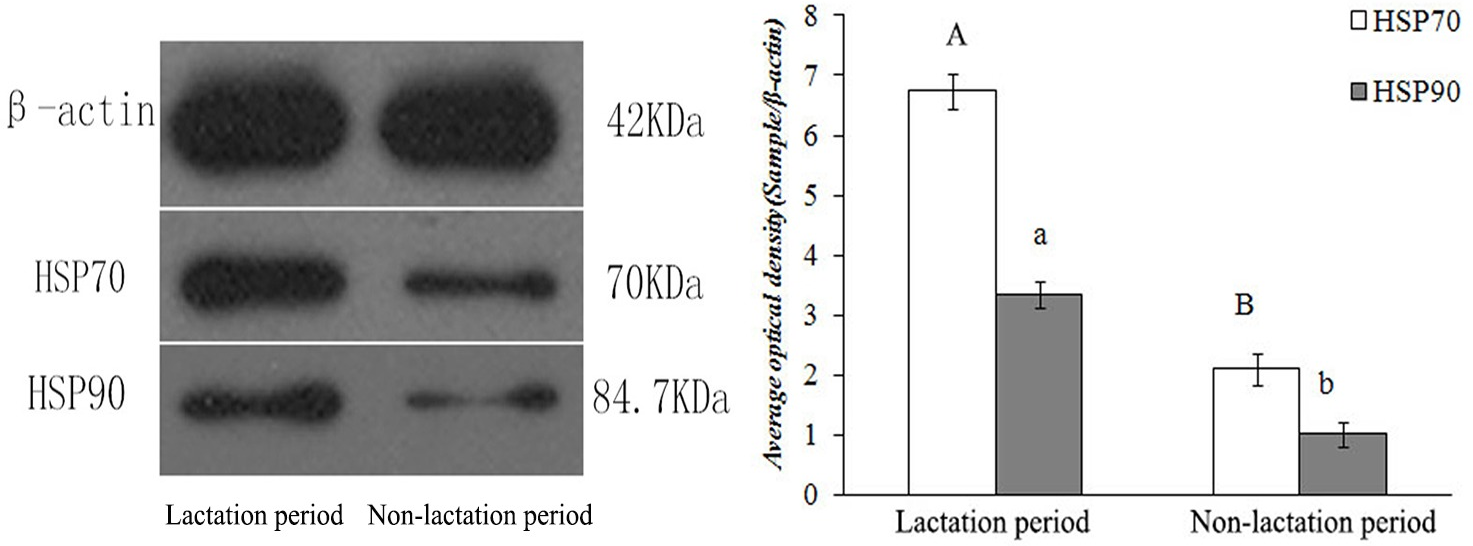
Protein expression levels of HSP70 and HSP90 detected using Western blotting analyses during the different lactation periods of female yaks. (A) Schematic representation of bands and size. (B) Comparative analysis of relative expression by integrated optical density.

The HSP70/90 gene and protein were expressed at different levels in tissues during lactation and non-lactation periods. As shown in (Figs 3C and 10), the highest expression level of HSP70/90 was in the lactation period. In contrast, the lowest expression level was in the non-lactation period. Breast tissue had especially high expression levels in the lactation period, which were over 3 times greater than in the non-lactation period. The expression level of HSP70 was higher than that of HSP90 in breast tissues, meanwhile, HSP70 and HSP90 showed significant differences in yaks during lactation and non-lactation periods (P<0.01), as shown in (Tables 2 and 3).

**Fig 11:**
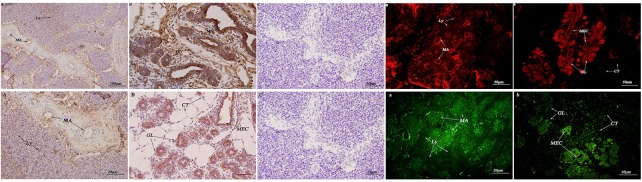
Immunohistochemistry and immunofluorescence localization of HSP70/90 in the breast of female yaks during different lactation periods. (A) Representative micrograph of a breast tissue section collected during a non-lactation period; positive staining for HSP70 was observed in the atrophic acinar and lymphocytes in the breast of *Bos grunniens*. (B) Representative micrograph of a breast tissue section collected during a lactation period; strong staining was observed in the mammary epithelial cells in the glandular lobules and connective tissue. (C) The control section collected during a non-lactation period from *Bos grunniens*, without immunoreactions (negative control). Mammary alveoli (MA); Lymphocyte (Ly); mammary epithelial cells (MEC); glandular lobules (GL); and connective tissue (CT).

As shown in (Fig 11), the expression levels of HSP70 in the connective tissue around the breast lobules were extremely strong in mammary epithelial cells, with no expression in the cytoplasm or nucleus. The expression levels of HSP90 in the breast lobules were remarkably strong in part of the epithelial cell membrane, and the expression in the nuclear membrane of the connective tissue nuclei was weak.

**Fig 12:**
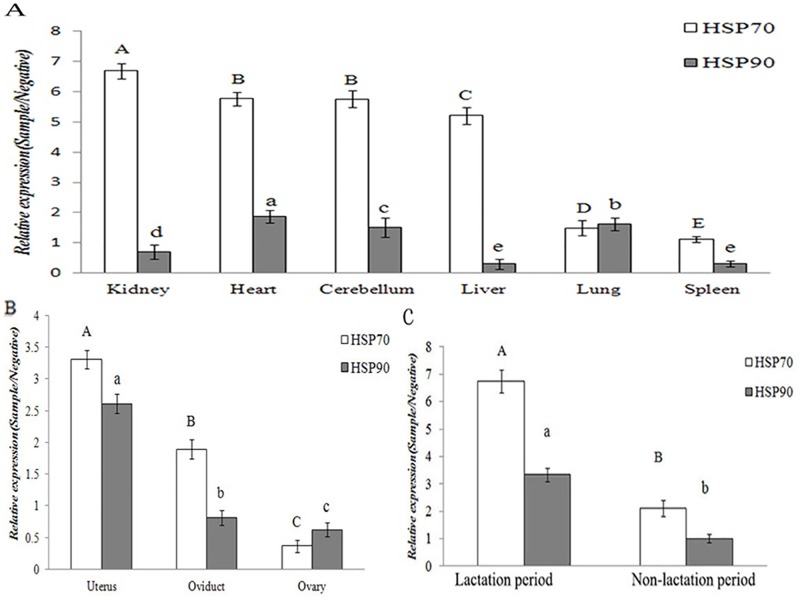
Immunohistochemistry and immunofluorescence optical density value of HSP70/90 in female yaks. Relative value (Sample/Negative). (A) Normal tissue; (B) the female reproductive system; and (C) the breast during different lactation periods.

The immunohistochemistry and immunofluorescence optical density values of HSP70/90 in female yaks are shown in (Fig 12). The results are consistent with the Western blot and quantitative fluorescence analyses.

## Discussion

This study isolated, sequenced and characterized cDNA clones that encoded HSP90 from yaks for the first time. Three ORFs present in the HSP90 sequence illustrated the frame-shifting property of the sequence. From research in eukaryotic cells, the upstream promoter sequence of the heat shock protein gene is TATA. A region of approximately 20 bp in size, which is known as the heat shock element (HSE) and is also necessary for the transcription of specific nucleotide sequences [14]. The nucleotide sequence homology of yak HSP90 and yak-cattle, cows, and goats is very high, which indicated that yaks have highly close evolution and genetic relationship with the above animals. The differences in the amino acid sequences might lead to differences in the protein spatial structures. In humans, HSP90 was rich in glutamine [12]. However, this study demonstrated that the yak HSP90 is rich in glutamate and lysine, and the results validated that the HSP90 gene can be used as a single nucleotide polymorphism marker to evaluate the loss of genetic diversity due to the conservative genetic properties of yaks.

Few studies have focused on the expression and localization of heat shock proteins 70/90 (HSP70/90) in yaks under normal physiological conditions. Analysis of the integrated optical density values showed that the HSPA2 protein expression levels in yak testes were the strongest, followed by the brain, kidney, heart, lung and liver, and its expression in spleen was the weakest [32]. With older piglets, decreased expression levels were also observed in the liver, lungs and kidney for HSC70 and in the kidney for HSP70 [33]. This study showed that the gene and protein expression level trends of HSP70/90 in tissues are similar, but there are differences between the expression levels of the two proteins. So, expression trends are basically identical in tissue besides mammary gland and testis. The highest expression levels of the HSP70 gene and protein were shown in the kidney, heart and cerebellum of yak. Therefore, this research suggested that the high expression level of HSP70 contribute to the organisms’ metabolism. HSP70 has been shown to protect against cerebral vascular atherosclerosis, myocardial injury, and inflammation from injury [34, 35]. Thus, considering these reports of myocardial and vascular injury of organisms with our finding in this study that there are high expression in the heart and cerebellum of yak, we speculated that HSP70 can protect cells and tissue from damage by diseases.

Conversely, the highest expression levels of HSP90 were demonstrated in the heart, cerebellum, liver and lung. According to reports in the literature, once porcine fetal fibroblasts were affected by cold stimulation, gene and protein expression levels of HSPs significantly increased [36]. Considering these reports in different tissues with our findings that the yaks received different stimuli under normal conditions, we speculated that the expression levels of HSP70/90 are regulated to protect animals. Previous researches found that different stimuli could induce significantly different HSPs expression levels in the bronchioles and respiratory bronchiole epithelial cells [37]. HSP70/90 are shown to play regulatory role in the adaptation to the high temperature and low salinity in marine environments [38]. This study demonstrated the localization of HSP70/90 in the cell membrane and cytoplasm of organs, but not the nuclei. Thus, cold, chronic hypoxia and metabolism increased the expression of HSP70/90. It can be proven that HSP70/90 play the active function in tissue physiological adaptation in yaks.

In this study, we determined that the expression levels of HSP70/90 were significantly different in the uterus, fallopian tube and ovary in the reproductive system of female yaks under normal physiological conditions (P<0.01). Gekle et al. [39] reported that the expression of HSP70 was high in the uterus, oviduct and all tissues of chick embryos, and it was higher in young embryos compared to mature embryos regardless of hot and cold stimulation; HSP70 participated the development of embryo. However, our study demonstrated high expression of HSP90 in the ovaries and uterus in female yaks. In additon, animal studies have previously shown that its expression levels in the uterine cervix and fallopian tube were significant during the luteal phase. When in the estrus period, the expression of HSP family members in the uterus, cervix, fallopian tube and other organs were obviously enhanced, and then promoted sperm movement in the uterus [40]. The growth of bovine preimplantation embryos and HSP expression were related to each other. Compared with normal newborns, the reduction in the expression of HSP in the placenta of premature births revealed that HSPs played role in maintaining embryo development during pregnancy [41]. The increasing expression level of HSP90 in the blastocysts cultured in vitro was associated with the developmental competence of the embryo [42]. In this study, we found that the HSP70/90 expression levels were higher in the non-reproductive system compared to the reproductive system of yaks. In addition, the weaker expression of HSP70/90 were shown in the female reproductive system with the luteal-phase of yaks. This finding suggested that HSP70/90 levels could increase within these tissues of the body due to the vigorous metabolism of the yaks.

Our study showed for the first time that HSP70/90 were localized in yak breast tissues. The expression levels of HSP70 in the connective tissue around the breast lobules were extremely strong in the mammary epithelial cell membranes, with no expression in the cytoplasm and nucleus. Numerous studies have demonstrated that lactation ability decreased in hot weather for sows. HSP70 was sensitive to heat stress and could reduce milk production in dairy cows [43, 44]. However, the expression levels of HSP90 in the breast lobules were highly strong in part of the epithelial cell membranes. The expression in the nuclear membrane of the connective tissue nuclei was weak. The research has also detected the expression of HSPA1 in the breast mammary epithelial cells, cardiac muscle cells, and renal tubules [45]. HSP27/70/90 were significantly increased when bovine mammary epithelial cells cultured in vitro were stimulated by temperature [46]. This phenomenon proved that the main location of HSP70/90 are in the mammary epithelial cells, and they are important factors to protect the epithelial cells of the mammary gland. This study also found that the expression levels of HSP70/90 in breast tissue during the lactation period are over 3 times those in the non-lactation period. The authors of this study believe that milking capacity and breast development increase the expression of HSP70/90, and HSP70/90 play the active function in the secretion of milk of yaks.

HSP70/90 are known to not only function biologically as molecular chaperone but also play important role in the activation of nuclear hormone, the maturation of signaling molecules, the formation of vesicles and the transportation of protein [47]. The expression of HSP70/90 were mutually coordinated at times. When encountering a target protein, HSP90 changed its shape, created a complex with HSP40 and worked synergistically with HSP70 [48]. However, the expression of HSP70 was higher than HSP90 in all examined tissues. The authors believe that HSP70/90 play role in the regulation of the breast for yaks in different lactation period.

Through quantitative expression and localization analysis of HSP70/90 in different visceral organs and tissues of female yaks, differences in the uterus, fallopian tube and ovaries, were observed. The expression levels of HSP70/90 were significantly different during the yaks’ lactation and non-lactation periods and were mainly located in mammary gland tissues. To further study the functional mechanisms of HSP70/90 during the lactation period, an experimental model is needed for the detection of HSP70/90 functions with single stimuli.

## Conclusions

The following conclusions can be drawn from this study. (1) The cDNA sequence of HSP90 was cloned, and a characteristic analysis of the gene was performed. HSP90 is the more highly conserved protein. (2) HSP70/90 have obvious differential expression in different tissues, with especially high expression in breast tissue during the lactation period. The expression levels of HSP70 are higher than those of HSP90 in all examined tissues. (3) Positioning analysis revealed that the HSP70/90 protein were mainly concentrated in the cell cytoplasm and partial cell membrane. (4) HSP70/90 play important role in tissue specificities of yaks as well as lactating function.
